# Editorial for the Special Issue on Development of CMOS-MEMS/NEMS Devices

**DOI:** 10.3390/mi10040273

**Published:** 2019-04-24

**Authors:** Jaume Verd, Jaume Segura

**Affiliations:** Electronic Systems Group (GSE), University of the Balearic Islands, E-07122 Palma (Illes Balears), Spain; jaume.verd@uib.eu (J.V.); jaume.segura@uib.es (J.S.)

Micro and nanoelectromechanical system (M/NEMS) devices constitute key technological building blocks to enable increased additional functionalities within integrated circuits (ICs) in the More-Than-Moore era, as described in the International Technology Roadmap for Semiconductors. The CMOS ICs and M/NEMS dies can be combined in the same package (SiP) or integrated within a single chip (SoC). In the SoC approach, the M/NEMS devices are monolithically integrated together with CMOS circuitry, allowing the development of compact and low-cost CMOS-M/NEMS devices for multiple applications (physical sensors, chemical sensors, biosensors, actuators, energy actuators, filters, mechanical relays, and others). On-chip CMOS electronics integration can overcome limitations related to the extremely low-level signals in sub-micrometer and nanometer scale electromechanical transducers, enabling novel breakthrough applications. In addition, nanoelectromechanical relays have been recently proposed for mechanical logic processing and other applications in CMOS–NEM hybrid circuits spreading the More-Than-Moore approach.

This Special Issue includes 11 papers dealing with the use of CMOS-M/NEMS devices not only in the field of sensing applications (infrared sensors, accelerometers, pressure sensors, magnetic field sensors, mass sensors) but also as clock references and integrated mechanical relays. The issue covers a wide range of topics inherent to these multidisciplinary devices related to fabrication technology, mechanical and functional characterization and interfacing with CMOS electronics design.

In particular, Göktas [1] analyzes theoretically and via FEM simulations the potential of using micromachined beam structures as ultra-sensitive CMOS-MEMS temperature sensors for infrared (IR) sensing applications. In the same topic, from a more experimental perspective, Duraffourg et al. [2] report on the fabrication and characterization of a dense array of nanoresonators, with a cross-section of 250 nm × 30 nm, whose resonant frequency changes with the incident IR-radiation, allowing temperature sensitivities down to 20 mK. The work by Miguel et al. [3] outlines a novel characterization method to determine the maximum deflection of the flexible top plate of a capacitive MEMS pressure sensor based on using an atomic force microscope in contact mode. The work by Lin and Dai [4] proposes a micromagnetic field sensor based on a magnetotransistor and four hall elements with the advantage of not requiring post-CMOS processing. The work by Liu et al. [5] reviews the sensing mechanisms, design, and operation of miniaturized MEMS gas sensors focusing on the monolithic CMOS–MEMS approaches. The work by Li et al. [6] proposes a high-precision miniaturized three-axis digital tunneling magnetic resistance-type sensor with a background noise of 150 pT/Hz^1/2^ at a modulation frequency of 5 kHz using an interface circuitry designed on a standard CMOS 0.35 µm technology. In the work of Perelló-Roig et al. [7], the design, fabrication, and electrical characterization of an electrostatically actuated and capacitive sensed 2-MHz plate resonator structure that exhibits a predicted mass sensitivity of ~250 pg·cm^−2^·Hz^−1^ is presented. The work of Riverola et al. [8] presents a tungsten seesaw torsional relay monolithically integrated in a standard 0.35 μm CMOS technology capable of a double hysteretic switching cycle, providing compactness for mechanical logic processing. Chan Jo and Young Choi [9] present a novel encapsulation method of NEM memory switches based on alumina passivation layers being fully compatible with the CMOS baseline process that allows locating NEM memory switches in any place, making circuit design more volume-efficient. Li et al. [10], reports on a high-order ΣΔ modulator circuit fabricated in a standard 0.35 μm CMOS process acting as a low-noise digital interface circuit for high-Q MEMS accelerometers. Finally, the work of Islam et al. [11] reports a real-time temperature compensation technique to improve the long-term stability of a ~26.8 kHz self-sustained MEMS oscillator that integrates a single-crystal silicon-on-insulator (SOI) resonator with a programmable and reconfigurable single-chip CMOS sustaining amplifier. 

We would like to warmly thank all the authors for publishing their works in this SI and specially to all the reviewers for dedicating their time and for helping to improve the quality of the submitted papers.
